# ‘Kitchen and cooking,’ a serious game for mild cognitive impairment and Alzheimer’s disease: a pilot study

**DOI:** 10.3389/fnagi.2015.00024

**Published:** 2015-03-17

**Authors:** Valeria Manera, Pierre-David Petit, Alexandre Derreumaux, Ivan Orvieto, Matteo Romagnoli, Graham Lyttle, Renaud David, Philippe H. Robert

**Affiliations:** ^1^EA CoBTeK/IA (Cognition Behavior Technology), Institut Claude Pompidou, University of Nice Sophia Antipolis, NiceFrance; ^2^Testaluna S.r.l., MilanoItaly; ^3^Kainos Evolve Ltd., BelfastUK; ^4^Centre Edmond et Lily Safra pour la Recherche sur la Maladie d’Alzheimer – Centre Méemoire de Ressources et de Recherche – Institut Claude Pompidou – CHU de Nice, NiceFrance

**Keywords:** serious game, Alzheimer disease, mild cognitive impairment, apathy, executive functions

## Abstract

Recently there has been a growing interest in employing serious games (SGs) for the assessment and rehabilitation of elderly people with mild cognitive impairment (MCI), Alzheimer’s disease (AD), and related disorders. In the present study we examined the acceptability of ‘Kitchen and cooking’ – a SG developed in the context of the EU project VERVE (http://www.verveconsortium.eu/) – in these populations. In this game a cooking plot is employed to assess and stimulate executive functions (such as planning abilities) and praxis. The game is installed on a tablet, to be flexibly employed at home and in nursing homes. Twenty one elderly participants (9 MCI and 12 AD, including 14 outpatients and 7 patients living in nursing homes, as well as 11 apathetic and 10 non-apathetic) took part in a 1-month trail, including a clinical and neuropsychological assessment, and 4-week training where the participants were free to play as long as they wanted on a personal tablet. During the training, participants met once a week with a clinician in order to fill in self-report questionnaires assessing their overall game experience (including acceptability, motivation, and perceived emotions). The results of the self reports and of the data concerning game performance (e.g., time spent playing, number of errors, etc) confirm the overall acceptability of Kitchen and cooking for both patients with MCI and patients with AD and related disorders, and the utility to employ it for training purposes. Interestingly, the results confirm that the game is adapted also to apathetic patients.

## Introduction

The term dementia indicates a decline in mental ability severe enough to interfere with activities of daily life (Dubois et al., 2010). Due to the increasing average lifespan, the occurrence of dementia has risen dramatically, thus engendering high socio-economic costs. For this reason, the early detection and the treatment of dementia are considered as a research priorities (Ballard et al., 2011). Dementia is characterized by the presence of cognitive symptoms, such impaired memory, attention, orientation and executive functions, which are often associated to behavioral and psychological symptoms, such as apathy or agitation (Aalten et al., 2003). Dementia can result from different causes, the most common being Alzheimer’s disease (AD), and it is often preceded by a predementia stage, known as mild cognitive impairment (MCI), characterized by a cognitive decline greater than expected for an individual’s age, but which does not interfere notably with activities of daily life (Petersen et al., 1999; Gauthier et al., 2006).

Serious games (SG) – which are digital applications specialized for purposes other than entertaining (such as educating, informing, or enhancing cognitive and/or physical functions) – are now widely recognized as promising non-pharmacological tools to help assess and evaluate patients’ functional impairments, as well as to help the patients’ treatment, stimulation, and rehabilitation (Robert et al., 2014). Boosted by the publication of a Nature letter showing that video game training can enhance cognitive control in older adults (Anguera et al., 2013), there is now a growing interest in developing SG specifically adapted to people with AD and related disorders. Preliminary evidence shows that SG can successfully be employed to train physical and cognitive abilities in people with AD, MCI, and related disorders. McCallum and Boletsis (2013) performed a literature review of the experimental studies conducted to date on the use of SG in neurodegenerative disorders. In summary, the results of the 15 reported studies suggest that: (1) physical games (or exergames, i.e., games that promote physical fitness) can positively affect several health areas of the players with mild AD and MCI, such as balance and gait (Padala et al., 2012), and voluntary motor control (Legouverneur et al., 2011); (2) cognitive games (i.e., games which target cognitive improvement) can improve a number of cognitive functions, such as attention and memory (Stavros et al., 2010; Weybright et al., 2010; Rosen et al., 2011) and visuo-spatial abilities (Yamaguchi et al., 2011); (3) both physical and cognitive games can have a positive impact on social and emotional functions, for instance they can improve the mood and increase positive affect and sociability (Weybright et al., 2010; Boulay et al., 2011; Yamaguchi et al., 2011) and reduce depression (Férnandez-Calvo et al., 2011).

Despite these promising results, a number of studies showed that elderly people and people with AD have problems in using many of the SG currently available on the market. Their difficulties include problems in getting familiar with the game technology and embarrassment about using the tools designed for the game (e.g., Wollersheim et al., 2010; Legouverneur et al., 2011). Furthermore, certain games were considered too demanding or even risky for elderly people (e.g., Sohnsmeyer et al., 2010). These difficulties derive from the fact that most of the SG currently employed have been developed for entertainment purposes (e.g., the Nintendo Wii Fit, Wii Sports, and Big Brain Academy) and with a “typical healthy user” in mind. To overcome this problem, SG targeting specifically AD and related disorders are starting to emerge (e.g., Benveniste et al., 2010; Nor Wan Shamsuddin et al., 2011; Tarnanas et al., 2013).

The purpose of the present paper is to report the results of a feasibility study conducted with patients with MCI and AD and related disorders with the game “Kitchen and cooking,” a SG game developed in the context of the European FP7 project VERVE (Vanquishing Fear through e-inclusion,http://verveconsortium.eu/). Kitchen and cooking is born from the tight collaboration between clinicians and game designers. Based on a recent survey showing that food is the most interesting topic for elderly people living in nursing homes (Leone et al., 2012), we developed the game based on a cooking plot, where participants can play different scenarios/recipes. Kitchen and cooking targets executive functions, specifically planning abilities, but includes also activities training attention and object recognition, as well as praxis. Following the recommendations of Robert et al. (2014) and Fua et al. (2013), the game can keep track of participants’ performance overtime, and thus can be employed also for assessment purposes. Furthermore, it takes into account the users’ impairments: for instance, after a number of errors, the user is helped with some prompts. Finally, it is installed on a tablet, which is an inexpensive and easy to use interface that can be employed anywhere.

In order to test how the SG is used in different environments, we included both outpatients and patients living in nursing homes. Furthermore, we included both apathetic and non-apathetic patients, as one challenges of the project VERVE was to develop new technologies to support the treatment of people with apathy associated to aging, or to neurological disorders.

## Materials and Methods

### Participants

Nine MCI patients (seven female and two male; mean age = 75.8 years; SD = 9.1; age range = 60–84) and 12 patients with AD or related disorders (eight female, four male; mean age = 80.3 years; SD = 6.3; age range = 70–90) voluntarily participated in this study. Patients were recruited either at the Nice Research Memory Center and CoBTeK research unit (CMRR), located at the Institut Claude Pompidou (MCI: *N* = 6, AD: *N* = 8) or in a nursing home working with the CMRR (MCI: *N* = 3, AD: *N* = 4). MCI diagnosis was conducted according to the National Institute on Aging and Alzheimer Association group clinical criteria (Albert et al., 2011), and the AD diagnosis was made according to the NINCDS ADRDA criteria (McKhann et al., 1984). Participants were not included if they had an active episode of major depression, if they had major perceptual (visual or auditory) impairments, rigidity or trembling (according to the UPDRS III; Fahn and Elton, 1987) or epilepsy. The mini mental state exam (MMSE) was used to evaluate the level of cognitive impairment for each group (Folstein et al., 1975). AD patients scored between 15 and 24 (*M* = 18.4, SD = 3.2) and MCI patients scored from 24 to 30 (*M* = 27.2, SD = 1.9). The presence of apathy was evaluated by means of the diagnostic criteria for apathy (Mulin et al., 2011), and the criteria have been used to divide the population in apathetic versus non-apathetic subjects. In addition, the severity of apathy was assessed using the Apathy Inventory – clinician version (Robert et al., 2009), a 12-point scale evaluating the presence of reduced initiation, interest, and emotional blunting.

Characteristics of MCI and AD subjects are presented in **Table 1**. The age, level of education, and gender distribution were not significantly different between the two groups. All participants gave their informed written consent before beginning the study. The study was performed in compliance with the Declaration of Helsinki, and was approved by the local ethics committees.

**Table 1:** Characteristics and group comparisons for mild cognitive impairment (MCI) and Alzheimer’s disease (AD) participants.

	MCI group (*N* = 9)	AD group (*N* = 12)	*p*
Female, *n* (%)	7 (77.8%)	8 (66.7%)	0.577
Age (years), mean ± SD	75.8 ± 9.1	80.3 ± 6.3	0.422
Level of education, *n* (%)			0.738
Primary education	2 (22.2%)	4 (33.3%)	
Secondary education	3 (33.3%)	3 (25.0%)	
Secondary education	2 (22.2%)	1 (8.3%)	
Higher education	2 (22.2%)	4 (33.3%)	
Residency, *n* (%)			1.000
Outpatients	6 (66.7%)	8 (66.6%)	
Nursing home	3 (33.3%)	4 (33.3%)	
MMSE, mean ± SD	27.2 ± 1.9	18.4 ± 3.2	0.000*
IADL-E, mean ± SD	5.8 ± 2.0	9.5 ± 4.0	0.028*
ADL, mean ± SD	2.1 ± 2.9	2.3 ± 2.0	0.917
TMT A (sec), mean ± SD	65.3 ± 41.0	176.4 ± 153.2	0.015*
Victoria Stroop Test (word/dot), mean ± SD	1.31 ± .35	1.78 ± .52	0.023*
Victoria Stroop Test (interference/dot) time, mean ± SD	1.93 ± .98	2.68 ± 1.29	0.129
VAT, mean ± SD	11.3 ± 1.3	7.9 ± 2.8	0.000*
Presence of diagnostic criteria for Apathy, *n* (%)	3 (33.3%)	8 (66.7%)	0.130
Apathy inventory, mean ± SD	1.8 ± 2.9	4.6 ± 2.5	0.041*

Group comparisons were made using Mann–Whitney *U* test (*p < 0.05) and chi-square (*p < 0.05) for categorical testing.

### Materials and Procedure

**FIGURE 1:**
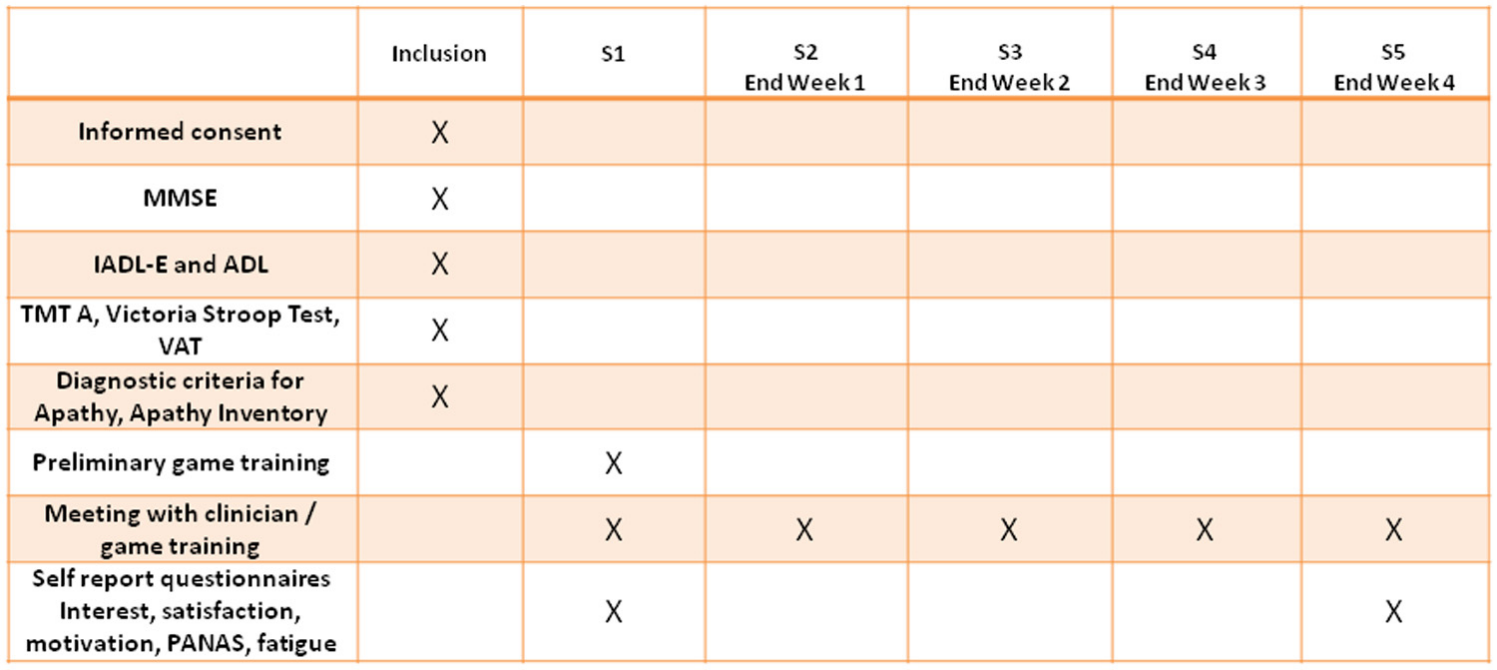
Flowchart summarizing the activities performed in the different experimental sessions.

A flowchart summarizing the study procedure with the different experimental sessions is reported in **Figure 1**. Participants performed a 4-week training with ‘Kitchen and cooking’ game installed on a tablet. During the training, participants were asked to play at home as much as they wanted, and they meet five times with a trained clinician. During the inclusion visit, after signing the informed consent, participants underwent the cognitive and functional assessment (see below). During the first session with the clinician (S1), they underwent a preliminary game training for Kitchen and cooking, in which the clinician showed all the ingredients/objects used in the different scenarios (in order to ensure that the objects were recognizable), and showed which gestures needed to be performed to complete the different game actions. Second, participants played one of the available scenarios in front of the clinician, who provided additional explanations (if necessary) in order to allow the participant to successfully complete the scenario. Finally, participants completed some self-report questionnaires to assess the game acceptability and interest. The homework for week 1 was to play as much as they wanted to the scenario seen in S1 (participants were allowed to try and play other scenarios, if they wanted). After 1-week participants meet again with the clinician (S2) and played with him the scenario played in S1, together with a new scenario, which represented the homework for week 2. The structure of S3 and S4 sessions was identical to that of S2, and every week participants exercised on a new scenario. During the last session with the clinician (S5), taking place at the end of week 4, participants played again the scenario performed during week 4, and they completed the same self-report questionnaires assessing the game acceptability and interest administered after S1.

### Cognitive and Functional Assessment

Global functioning was evaluated during the inclusion visit by means of the MMSE, the Instrumental Activities of Daily Living scale (IADL; Mathuranath et al., 2005) and the Independence in Activity of Daily Living index (ADL; Katz et al., 1970). Attention and mental flexibility were measured with the Trail-Making Test, versions A (Lezak et al., 2004). Executive functions (specifically, selective attention, and inhibition control) were evaluated using the interference scores from the Victoria Stroop Test (Word/Dot and Interference/Dot, Bayard et al., 2011). Memory performance was evaluated by means of the Visual Association Test (VAT; Lindeboom et al., 2002).

### ‘Kitchen and Cooking’ Game

“Kitchen and cooking” game is a SG developed in the context of the European FP7 project VERVE (Vanquishing Fear through e-inclusion, http://verveconsortium.eu/), a project which aimed to develop new technologies to support the treatment of people at risk of social exclusion, either because of fear and apathy associated with aging, or because of a neurological disorder. The CMRR and the CoBTeK team designed the cognitive task embedded in the game. The game was developed by Testaluna s.r.l. (Milan, Italy), and Kainos Evolve Ltd (Belfast, UK) developed the game interface used by the clinicians.

Kitchen and cooking is based on a cooking plot, where participants can play four different scenarios/recipes: pizza, yogurt cake, chicken breast in cream sauce, and finally salmon wrap. In each scenario, participants need: (a) to select the correct ingredients from the fridge and cupboards, a searching task with engages object recognition and sustained attention (*gnosis* activity); (b) to plan which actions need to be performed, and in which order, a task requiring planning abilities (*executive functions* activity); and finally, to perform specific gestures to accomplish each action (e.g., to rotate the finger to mix the ingredients), a task which requires praxis abilities (*praxis* activity). Depending on the scenario, the number of objects to be recognized ranges from 5 to 7, the number of executive functions activities ranges from 5 to 8, and the number of praxis ranges from 7 to 13.

The game can keep track of the time spent playing a scenario and of the time spent on each of the game activities (gnosis, executive functions, and praxis), of the total number of scenarios played (successfully completed or not), and of the number of errors performed in each game activity.

A demo showing Kitchen and cooking can be seen on the website of the Association Innovation Alzheimer, at the following link: http://www.innovation-alzheimer.fr/projets-en/verve-en/

### Self Report Questionnaires

At the end of S1 and S5 participants were administered self-report questionnaires concerning the game experience. Specifically, the perceived satisfaction was assessed through a 10 cm analogical scale, in which participants were asked to bisect a line ranging from ‘not satisfied at all’ to ‘really satisfied’ in order to indicate their degree of satisfaction for the game. Perceive interest was assessed through a 4-item 1–7 Likert scale adapted from Gourlan et al. (2013). Similarly, motivation was evaluated through an adaptation of the scale proposed by Gourlan et al. (2013), a 24-item 0–7 Likert scale which measures separately intrinsic motivation (e.g., “I play because it is fun”) and external motivation (“I play because my friends/family members say I should”). Experienced emotions were assessed through the PANAS scale (Watson et al., 1988), a 20-item 0–5 Likert scale evaluating separately self-reported positive and negative emotions. Finally, fatigue was evaluated through the French adaptation of the Piper Fatigue Scale (11 rating questions, scale 0–10; Gledhill et al., 2002).

### Statistics Analyses

All statistical analyses were computed using SPSS 20.0. In order to verify the acceptability of the intervention, we computed: (a) the number of participants that successfully completed the training; (b) the mean scores of the acceptability questionnaires administered after S1 and S5; (c) the mean time spent playing, and the number of scenarios played (in total and at home). Group comparisons were performed using diagnosis (MCI vs. AD), residence (outpatients vs. patients in nursing home) and presence of diagnostic criteria for apathy (yes vs. no) as independent between-subject factors. As the distribution of the data was not normal, group comparisons were performed using non-parametric Mann–Whitney *U* test. Comparisons between the acceptability questionnaires after S1 and S5 were performed using paired-sample *t*-tests.

In order to verify whether the assessment of the different game activities (gnosis, executive functions, and praxis) was in line with the classical functional and neuropsychological assessment, we computed for each participant the mean time spent to complete a scenario, and the errors and mean time spent on each game activity in S1 (first session), and we submitted them to separate Mann–Whitney *U* test, with diagnosis (MCI vs. AD), residence (outpatients vs. patients in nursing home) and presence of diagnostic criteria for apathy (yes vs. no) as independent between-subject factors. We also compute correlations between the time spent on each game activity in S1 and the classical cognitive and functional assessment using Pearsons’ correlation coefficient (two-tails).

Finally, in order to verify if game activities improved with training, for each scenario we compared the time spent to perform each activity (gnosis, executive functions, and praxis) during the first session (S1, S2, S3, or S4 depending on the scenario and participant) and the next session (1 week after) using paired-sample *t*-tests. Group comparisons on the improvement scores (difference in time spent on an activity between two consecutive sessions) were performed with Mann–Whitney *U* test using diagnosis (MCI vs. AD), residence (outpatients vs. patients in nursing home) and presence of diagnostic criteria for apathy (yes vs. no) as independent between-subject factors. We also computed Pearsons’ correlations between game improvement scores and the total time spent playing during the training.

## Results

### Cognitive, Functional, and Behavioral Assessment

Demographic, cognitive, and functional characteristics of the patients are presented in **Table 1**. Compared to MCI participants, AD participants had significantly lower MMSE scores (*p* < 0.001) and IADL-E (*p* = 0.028), confirming the presence of a significant impairment in the activities of daily living. Furthermore, participants were slower at the TMT A (*p* = 0.015), more sensitive to interference in the Victoria Stroop Test – Word/Dot index (*p* = 0.023), and scored lower at the VAT memory task (*p* < 0.001) compared to MCI participants. Finally, AD participants had a higher Apathy Inventory compared to MCI participants (*p* = 0.041). However, no significant difference between groups was found concerning the presence of diagnostic criteria for apathy (χ2 = 2.29, *p* = 0.130). No significant difference between groups was found for the ADL, and the Victoria Stroop Test – Interference/Dot index.

### Intervention Acceptability

#### Training Completion and Self-Report Questionnaires

The 4-weeks training was successfully completed by 20 out of 21 participants (one participant abandoned the study after the first week). The results of the self-report questionnaires (mean scores between S1 and S5) showed that, as a group, participants reported to be highly satisfied concerning the game experience (Mean = 8.2/10, SD = 1.3), interested by the game (Mean = 17.1/28, SD = 5.6), and motivated by the activity. Specifically, intrinsic motivation (Mean = 3.9/7, SD = 1.3), was significantly higher than external motivation [Mean = 2.5/7, SD = 1.2; *t*_(15)_ = 4.37, *p* = 0.001]. Furthermore, participants reported to be not very fatigued (Mean = 3.7/10; SD = 1.2), and to have experienced more positive emotions (PANAS pos, *M* = 2.7/5; SD = 0.8) than negative emotions [PANAS neg, *M* = 1.4/5, SD = 0.6, *t*_(18)_ = 5.86, *p* < 0.001]. The interest, satisfaction, motivation (intrinsic and extrinsic) fatigue, and experienced emotions (positive and negative) did not change from S1 to S5 (*t* ranging from 0.15 to 1.8, *p*s ranging from 0.092 to 0.880), thus confirming the overall positive evaluation of the game also after a repeated training.

Results according to diagnosis, residence, and presence of diagnostic criteria for apathy are shown in Table 2. Group comparisons revealed that AD participants reported to be significantly more satisfied compared to the MCI participants (*p* = 0.043). Furthermore, apathetic participants reported to experience fewer positive emotions (*p* = 0.008) compared to non-apathetic participants. No difference in the self-report scales was found between outpatients and patients living in nursing homes (all *p*s > 0.323).

**Table 2:** Intervention acceptability.

	MCI (*N* = 9)	AD (*N* = 12)	Outpatients (*N* = 14)	Nursing home (*N* = 7)	Apathetic (*N* = 11)	Non-apathetic (*N* = 10)
Satisfaction, scale 0–10 (mean ± SD)	**7.6 (1.2)**	**8.6 (1.3)**	8.4 (1.4)	8.0 (1.3)	8.1 (1.4)	8.3 (1.3)
Interest scale 0–28 (mean ± SD)	18.3 (5.6)	16.0 (5.7)	17.1 (5.8)	17.1 (5.8)	17.3 (6.2)	16.9 (5.2)
Intrinsic motivation scale 1–7 (mean ± SD)	3.6 (1.4)	4.3 (1.1)	3.9 (1.2)	4.1 (1.6)	4.4 (1.1)	3.5 (1.3)
External motivation scale 1–7 (mean ± SD)	2.3 (1.4)	2.7 (1.0)	2.5 (1.0)	2.3 (1.9)	2.9 (1.3)	2.1 (1.1)
PANAS positive emotions scale 1–5 (mean ± SD)	2.6 (0.9)	2.8 (0.7)	2.8 (0.8)	2.5 (0.7)	**2.3 (0.6)**	**3.1 (0.8)**
PANAS negative emotions scale 1–5 (mean ± SD)	1.4 (0.7)	1.4 (0.6)	1.4 (0.5)	1.6 (0.9)	1.5 (0.8)	1.4 (0.5)
Fatigue scale 0–10 (mean ± SD)	3.8 (1.1)	3.5 (1.3)	3.6 (1.3)	3.8 (1.1)	3.7 (1.1)	3.7 (1.3)
Number of scenario played (mean ± SD)	54.1 (49.3)	57 (76.8)	**72.2 (74.7)**	**22.9 (9.6)**	74 (85.3)	35.7 (21)
Total time played (mean ± SD)	05h09m (04h12 m)	05h33m (04h34m)	06h18m (04h59m)	03h31m (01h26m)	**07h18m (05h04m)**	**03h16m (01h49m)**

*Results of the self report questionnaires (mean between S1 and S5), number of scenarios and total time played by MCI vs. AD patients, outpatients vs. patients living in nursing homes, and by apathetic vs. non-apathetic patients according to the Apathy diagnostic criteria. Results in bold indicate a significant difference at the Mann–Whitney *U* test (*p* < 0.05)*.

#### Game Experience

The acceptability of the intervention was corroborated also by the data concerning the time that patients spent playing, and the number of scenarios played. During the 4-week trial participants played a mean of 55.8 scenarios (SD = 64.9; range = 10–284), for a mean playtime of more than 5 h (5h22m; SD = 4h19m; range = 32m–17h40m), corresponding to a mean of 1h21m hours per week. Almost 85% of the scenarios were played at home (Mean = 47.4, SD = 64.3), for a mean playtime at home of 3h48m (SD = 4h19m range = 0m–16h28m). Over 70% of the scenarios were successfully completed (Mean = 70.2%, SD = 25%; range = 18.2%–100%).

Results according to diagnosis, residence, and presence of diagnostic criteria for apathy are shown in **Table 2**. No significant difference in the number of scenarios (*p* = 0.422) or time played (*p* = 0.808) was found between MCI and AD participants. Outpatients played more scenarios compared to patients in nursing home (*p* = 0.031), but the difference in the time spent playing did not reach statistical significance (*p* = 0.224). Interestingly, apathetic patients played longer than non-apathetic patients (*p* = 0.016), while no difference in the number of scenarios played was found (*p* = 0.654).

### Game Assessment

#### Time for Scenario Completion, Gnosis, Executive Functions, and Praxis

Results according to diagnosis, residence and presence of diagnostic criteria for apathy are shown in **Table 3**. AD participants took significantly longer to complete a scenario compared to MCI participants (*p* = 0.004). Furthermore, the first time a scenario was played with the clinician (*t*0), AD participants were significantly slower in the gnosis (*p* = 0.002), executive functions (*p* = 0.046), and praxis activities (*p* = 0.006) compared to MCI participants, and made more errors in the praxis activity (*p* = 0.046) thus suggesting that the game assessment was sensitive to differences in the level of general cognitive impairment. AD participants were significantly slower than MCI participants also when the scenario was played again with the clinician after 1 week of training at home (*t*1; gnosis time, *p* = 0.002; executive functions time, *p* = 0.003; praxis time, *p* = 0.004). No difference in the errors was found (all *p*s > 0.056). No difference in the mean time spent to complete a scenario, gnosis, executive functions, and praxis time/errors was found between outpatients and patients living in nursing homes (all *p*s > 0.157), nor between apathetic and non-apathetic participants (all *p*s > 0.175).

**Table 3:** Game assessment.

	MCI (*N* = 9)	AD (*N* = 12)	Outpatients (*N* = 14)	Nursing home (*N* = 7)	Apathetic (*N* = 11)	Non-apathetic (*N* = 10)
Scenario duration (mean ± SD)	**7m26s (2m51s)**	**11m44s (2m56s)**	9m21s (3m50s)	10m59s (2m53s)	10m28s (3m25s)	9m15s (3m47s)
Gnosis time *t*0 (mean ± SD)	**1m35s (0m48s)**	**3m06s (1m02s)**	2m20s (1m05s)	2m35s (1m28s)	2m45s (1m12s)	2m01s (1m07s)
Gnosis time *t*1 (mean ± SD)	**1m07s (0m30s)**	**2m52s (1m41s)**	1m41s (1m05s)	2m50s (2m03s)	2m18s (1m47s)	1m49s (1m14s)
Executive functions time *t*0 (mean ± SD)	**3m25s (1m04s)**	**4m37s (1m22s)**	4m14s (1m22s)	3m46s (1m24s)	4m16s (1m11s)	2m51s (1m36s)
Executive functions time *t*1 (mean ± SD)	**2m13s (1m11s)**	**4m31s (1m36s)**	3m26s (2m00s)	3m34s (1m35s)	3m44s (1m38s)	3m10s (2m05s)
Praxis time *t*0 (mean ± SD)	**3m13s (0m49s)**	**4m25s (1m02s)**	3m47s (1m06s)	4m04s (1m10s)	4m04s (1m00s)	3m39s (1m14s)
Praxis time *t*1 (mean ± SD)	**2m22s (0m35m)**	**4m07s (1m21m)**	3m10s (1m19s)	3m37s (1m32s)	3m37s (1m27s)	2m58s (1m16s)

*Mean time to complete a scenario, and mean time spent on each game activity in *t*0 (first time a scenario was played with the clinician) and *t*1 (1 week later) for MCI vs. AD patients, outpatients vs. patients living in nursing homes, and by apathetic vs. non-apathetic patients. Results in bold indicate a significant difference at the Mann–Whitney *U* test (*p* < 0.05)*.

#### Correlations Between Classical Cognitive Assessment and Game Assessment

The game gnosis time in S1 showed a significant correlation with the MMSE [*r*_(20)_ = -0.68, *p* = 0.001], the TMT A time [*r*_(20)_ = 0.59, *p* = 0.006], thus confirming that the object search and selection task can be considered a good proxy for attentional processes. As expected, the game executive functions time showed a significant correlation with the Victoria Stroop Test [Word/Dot index, *r*_(20)_ = 0.70, *p* = 0.001; Interference/Dot index, *r*_(20)_ = 0.55, *p* = 0.013], an index of inhibition control. Finally, the praxis time correlated significantly with the MMSE [*r*_(20)_ = -0.53, *p* = 0.016] and with both indexes of the Victoria Stroop Test [Word/Dot index, *r*_(20)_ = 0.71, *p* < 0.001; Interference/Dot index, *r*_(20)_ = 0.60, *p* = 0.005]. No other significant correlation was found (all *p*s > 0.153).

#### Improvement in the Game Activities During the Training

**FIGURE 2:**
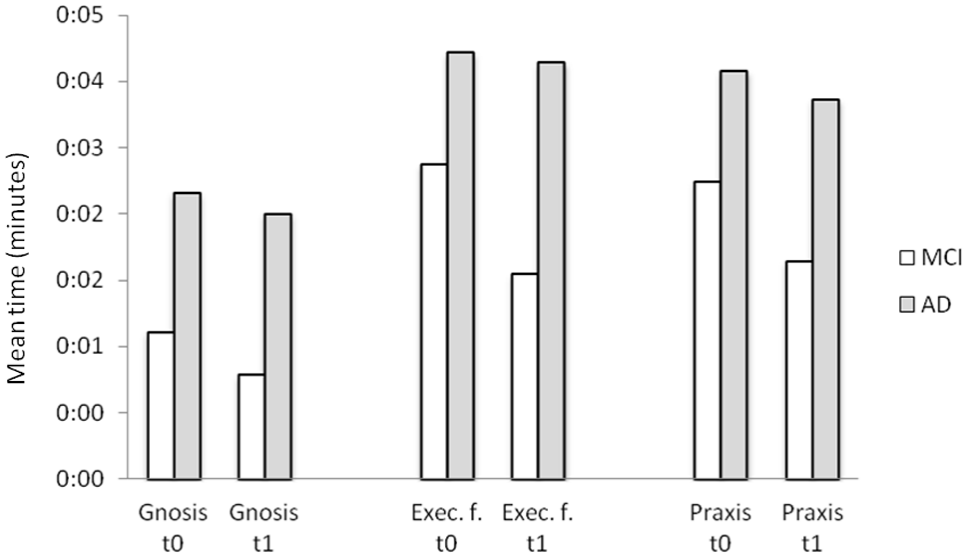
Mean time spent on the different game activities the first time a scenario was played (t0) and one week after (t1) for MCI and AD patients.

Every scenario was played with the clinician twice: the first time to practice the recipe (*t*0, in S1, S2, S3, or S4 depending on the scenario and participant), and the second time 1 week later (*t*1), before practicing another recipe. As the scenarios differ in length and complexity, we could not compare performance in S1 and S5, and we compared instead, for each scenario, performance in *t*0 and *t*1. Comparisons between the game assessment made during *t*0 and *t*1 revealed no differences in the gnosis time (*p* = 0.115), but a significant reduction is praxis and executive function time, with participants becoming faster in the praxis (*p* = 0.001) and executive functions activities from the practice to the follow up round (*p* = 0.017). No difference in the number of errors was found for any of the activities (all *p*s > 0.519). The improvement in the executive functions was greater for MCI compared to AD patients. Specifically, MCI participants showed a more consistent reduction in the time spent in the activity (*p* = 0.010; see **Figure 2**) and in the mean number of errors (*p* = 0.025) in *t*1 vs. *t*0 compared to AD participants. Furthermore, outpatients improved in the gnosis (reduction of time from *t*0 to *t*1) more than patients living in nursing home (*p* = 0.14). No significant difference between apathetic and non-apathetic patients was found.

A significant correlation was found between the improvement in the gnosis and the praxis [*r*_(20)_ = 0.55, *p* = 0.013], and between the praxis and the executive functions [*r*_(20)_ = 0.68, *p* = 0.001], thus suggesting that participants that became faster in the gnosis and executive functions became also faster in the praxis. The correlation between gnosis and executive functions did not reach statistical significance [*r*_(20)_ = 0.43, *p* = 0.057].

A significant correlation was found between the time spent playing during the 4-week training and the improvement shown in the gnosis [*r*_(20)_ = 0.52, *p* = 0.020] and executive functions time [*r*_(20)_ = 0.46, *p* = 0.040], thus suggesting that gnosis and executive functions (as assessed by the game) could be improved by exercise. The correlation between time spent playing and improvement in the praxis time was in the same direction, but did not reach statistical significance [*r*_(20)_ = 0.36, *p* = 0.124].

## Discussion

The results of the present feasibility study confirm that Kitchen and cooking was acceptable and interesting for both patients with MCI and AD. This interpretation is confirmed by the fact that 20 out of 21 participants successfully completed the 4-week training, and by the fact that participants rated the game experience as interesting, reported to be highly satisfied and motivated by the game, to experience more positive emotions than negative emotions, and not to be fatigued both at the beginning and at the end of the training. Moreover, participants played a mean of almost one and a half hours per week (corresponding to 14 scenarios), thus suggesting that they played also outside the meetings with the clinician. Interestingly, there was a huge variability in the playing time: while a few participants played almost only with the clinician, some others played up to 70 scenarios per week, thus suggesting that the game most probably met their interest, and was particularly adapted to them. This variability in the playing time confirms that adaptation to the patients’ interest and level impairment is a key challenge in designing SGs with training purposes (Robert et al., 2014).

We are convinced that a critical factor in the success of the intervention was the presence of the clinician. We designed Kitchen and scenario as a tool to help the patient to train outside the clinical consultation, but the periodic supervision of the clinician is necessary to explain the functioning to the patient and the family, keep track of the evolution of the performance, to adapt the intervention step by step to the patients’ changing needs and to maintain the motivation.

Another interesting finding was that we found a significant difference in the number of scenarios played depending of the place of residence, with outpatients playing significantly more compared to patients in nursing home. The proportion of AD/MCI was not significantly different between outpatients and patients living in nursing home, suggesting that the level of impairment was similar in the two groups, and thus was not the critical variable in explaining the effect. One possible explanation concerns the level of initial engagement/commitment to the training. Outpatients needed to come to the Memory consultation five times during the 4-week training, which implies that they were very committed when they decided to take part in the study. On the contrary, for patients living in nursing homes the trainings with the clinician took place in the nursing home, which makes possible that some participants accepted even if they had a lower commitment. Another possibility is that outpatients played more because they were followed more closely by a family caregiver. The level of external motivation reported at the beginning and at the end of the training (e.g., “I play because my friends/family say I should”) did not differ between the two groups. However, it is possible that the simple sharing experience stimulated patients to play more.

The major limitation of these results is the small number of participants included in the study. The study was designed as a pilot experiment most specifically oriented to a feasibility target. In addition, it was important in order to fit the European commission requirement to include both outpatients and patients living in nursing home. This is obviously of interest, but it increases the heterogeneity of the population.

### A Serious Game for Apathy

Apathy is one of the most common neuropsychiatric symptoms of AD and related disorders, occurring in almost 65% of dementia patients (Ferri et al., 2005; König et al., 2014). Apathy is associated with a higher degree of global functional impairment (Doron et al., 2013) and therefore to a loss of autonomy in activities of daily living (Boyle et al., 2003; Robert et al., 2009). One of the challenges of the project VERVE was to design SG that, due to their playful nature, may be particularly adapted to target apathetic patients. The results of this feasibility study suggest that Kitchen and cooking was adapted to apathetic participants. Indeed, apathetic participants reported to be as interested, motivated, and satisfied by the game experience as non-apathetic participants, a result which in interesting on its own. Critically, apathetic patients played *more* during the 4-week training compared to non-apathetic patients, suggesting that they were not impaired in this specific goal-directed activity. At a first glance, this may seem counterintuitive. A possible explanation concerns the fact that non-apathetic participants have more interests and external activities compared to apathetic participants, and thus had less time that they wish to dedicate to the game. Future studies including qualitative interviews may be useful to corroborate this interpretation.

### Kitchen and Cooking as an Assessment Tool

Correlation analysis revealed that performance in the different game activities was consistent with performance in the classical functional and neuropsychological tests. Specifically, the game gnosis time at the beginning of the training correlated with the TMT A time, thus confirming that the object search and selection task can be considered a good proxy for attentional processes, and the game executive functions time showed a significant correlation with the Victoria Stroop Test, an index of inhibition control. Furthermore, AD participants spent more time to complete a scenario compared to MCI participants, and were slower in the gnosis, praxis, and executive functions time, thus suggesting that the assessment made with Kitchen and scenario was in line with that made using classical assessment instruments.

This SG was not designed to substitute the classical functional, behavioral, and neuropsychological assessment. However, the present results suggest that it may be useful to complement classical assessment methods. For instance, it could be easily employed to track the evolution of executive functions and attentional deficits overtime. Furthermore, due to its playful nature, it may be particularly adapted to patients whose performance is strongly influenced by test anxiety. For instance a heavily impaired performance at the classical tests associated to preserved functioning in the SG activity may prompt the clinician to be more cautious in the interpretation of the test results.

### Kitchen and Cooking a Training Tool

The results of the training suggest that performance in the different game activities could be improved overtime. After 1 week of practicing on a scenario, participants became faster in both executive functions activity and praxis activity. Our training was designed to test feasibility and not the improvement in performance, so we did not ask participants to play every scenario in the first (S1) and last session (S5) with the clinician, meaning that we were unable to quantify exactly the improvement observed over 4 weeks. However, as patients were able to improve significantly their performance in 1 week, it is likely that the improvement in performance between S1 and S5 would be even more pronounced. This hypothesis is supported by the finding that participants who played more improved more in the game activities, specifically in the gnosis and executive functions.

Interestingly, MCI participants improved significantly more in the executive functions activity compared to AD participants. These results supports the view that early interventions targeting predementia stage are more effective in training cognitive abilities such as executive functions (REF), and suggest that Kitchen and cooking (employed as a training tool) may be specifically adapted to people with MCI.

## Conclusion

Kitchen and cooking is a SG game developed with the tight collaboration between clinicians and game designers in the context of the European FP7 project VERVE. This study suggests that Kitchen and cooking was acceptable, interesting and motivating for both patients with MCI and AD, and that it was adapted also to apathetic participants. This suggests that Kitchen and cooking could be an additional tool for clinicians in order to stimulate apathetic patients. Given these promising results, we are going to use the game in clinical practice, and propose the game to the patients coming for consultation to our Memory clinic and to the patients in the day centers, with a special focus on apathetic patients showing a loss or reduction in self-initiated behaviors, but preserved environmental-stimulated behaviors (Robert et al., 2009). This will allow us to collect additional data on the usability and acceptability of the game, and on its efficacy over longer training periods. Also, in order to allow the patients to select among a variety of activities, and to meet the interests of a wider variety of patients, we aim at creating new SGs.

## Conflict of Interest Statement

The authors declare that the research was conducted in the absence of any commercial or financial relationships that could be construed as a potential conflict of interest
